# Impact of unknown incidental findings in PET/CT examinations of patients with proven or suspected vascular graft or endograft infections

**DOI:** 10.1038/s41598-021-93331-4

**Published:** 2021-07-02

**Authors:** Lars Husmann, Nadia Eberhard, Martin W. Huellner, Bruno Ledergerber, Anna Mueller, Hannes Gruenig, Michael Messerli, Carlos-A. Mestres, Zoran Rancic, Alexander Zimmermann, Barbara Hasse

**Affiliations:** 1 Department of Nuclear Medicine, University Hospital Zurich, University of Zurich, Raemistrasse 100, 8091 Zurich, Switzerland; 2 Division of Infectious Diseases and Hospital Epidemiology, University Hospital Zurich, University of Zurich, Zurich, Switzerland; 3 Clinic for Cardiac Surgery, University Hospital Zurich, University of Zurich, Zurich, Switzerland; 4 Clinic for Vascular Surgery, University Hospital Zurich, University of Zurich, Zurich, Switzerland

**Keywords:** Infectious diseases, Cardiovascular biology

## Abstract

Vascular graft or endograft Infections (VGEI) are rare but severe complications of vascular reconstructive surgery, and associated with significant mortality and morbidity risk. Positron emission tomography/computed tomography with ^18^F-fluorodeoxyglucose (PET/CT) has been shown to have a high diagnostic accuracy in the detection of VGEI. In this single-center prospective cohort study, we assessed the rate and the impact on patient management of relevant unknown incidental findings in PET/CT of patients with proven or suspected VGEI, and clinical follow-up of all patients was performed. Our study results show a comparably high rate of relevant unknown incidental findings (181 in 502 examinations), with documented direct impact on patient management in 80 of 181 (44%) of all findings. PET/CT scan- and patient-based evaluation revealed impact on patient management in 76 of 502 (17%) of all PET/CT scans, and in 59 of 162 (36%) of all patients, respectively. Furthermore, PET/CT correctly identified the final diagnosis in 20 of 36 (56%) patients without VGEI. In conclusion, in proven and suspected VGEI, PET/CT detects a high rate of relevant unknown incidental findings with high impact on patient management.

## Introduction

Vascular graft or endograft Infections (VGEI) are a rare but severe complication of vascular reconstructive surgery, and are associated with a significant mortality and morbidity risk^1–3^. The therapy of VGEI is usually demanding and needs to be individualized with respect to clinical findings, graft material, site of infection, microorganism/s involved and patient co-morbidities. A combination of the overall appraisal of clinical presentation, laboratory, and imaging is considered the gold standard for the diagnosis of VGEI^4^. With respect to imaging, ^18^F-fluorodeoxyglucose (FDG) positron emission tomography/computed tomography (PET/CT) has been demonstrated to be highly accurate for the initial diagnosis^5–9^, and its usefulness in therapy control of VGEI has also been documented by initial studies^10,11^. Likewise, PET/CT has been shown to be accurate and sensitive in the detection of other infections and numerous malignant diseases^12–20^. Furthermore, as PET/CT is usually performed as a whole-body examination, a higher incidence of incidental findings may be expected compared to traditional imaging modalities, such as contrast-enhanced CT, which are usually limited to the anatomical site of a suspected VGEI in the chest and/or abdomen. Hence, PET/CT may potentially increase the likelihood of detection of hitherto unknown co-morbidities.

Therefore, the aim of our study was to determine the rate and the impact on patient management of unknown incidental findings in PET/CT examinations of patients with proven or suspected VGEI.

## Methods

### Study design

All patients were prospectively enrolled as part of the Vascular Graft Cohort Study (VASGRA; clinical trials. gov identifier: NCT01821664), an open, prospective observational cohort study^21^, including patients aged ≥ 18 years with any type of vascular graft operation and clinical suspicion of VGEI. The diagnosis of VGEI was defined as suggested by the Management of Aortic Graft Infection Collaboration (MAGIC). Every clinical case was adjudicated by a multidisciplinary team of infectious disease specialists, cardiovascular surgeons, radiologists, nuclear medicine physicians and microbiologists. The gold standard for VGI diagnosis was a positive microbiological culture of the deep tissue around the vascular prosthesis, obtained by open biopsy or a positive microbiological culture of an explanted vascular graft. In cases with excluded VGEI, clinical, laboratory, histopathological and imaging results were also considered, and served as the standard of reference.

A case file review was performed in all patients, and information collected at the time of the initial diagnosis, at the time of imaging and at the last clinical visit (recorded until June 2020). Data included patient demographics, laboratory data, results of microbiology, clinical information, and information about treatment. Written informed consent was obtained from all participants. The institutional ethics committee approved the study, namely the Kantonale Ethikkomission Zürich (KEK-ZH-Nr. 2012-0583). All procedures were performed in accordance with the 1964 Helsinki declaration and its later amendments or comparable ethical standards.

### PET/CT data acquisition

Patients fasted for at least four hours prior to PET/CT imaging. Blood glucose levels < 12 mmol/l were accepted^22^. The standardized uptake time was 60 min in supine position. A non-enhanced CT scan for attenuation correction and anatomic localization of 18F-FDG uptake was performed with arms overhead whenever possible, using automated dose modulation (range 10–100 mA, 120–140 kV) with a scan range from the vertex of the skull to the mid of the thighs or to the feet. Two types of PET/CT scanners were used within the study period between 2012 and 2020 (i.e. Discovery VCT, and Discovery MI, both GE Healthcare, Waukesha, WI), using either the 3-dimensional acquisition mode with a fixed scan duration of two minutes per bed position (Discovery VCT), or a time-of-flight acquisition mode with a fixed scan duration of 2.5 min per bed position (Discovery MI).

### PET/CT image analysis and definitions

All PET/CT were analysed independently on a dedicated workstation (Advantage Workstation, version 4.7, GE Healthcare Biosciences, Pittsburgh, PA) by two dually board-certified radiologists and nuclear medicine physicians, both with more than 8 years experience in hybrid imaging. Primary and secondary (e.g. infectious foci not in the vicinity of the aorta or other relevant findings such as malignancies) diagnoses were documented if deemed potentially relevant for patient management. A consensus reading was performed if results differed. All findings were retrospectively analysed, whether the findings were previously known (e.g.: by previous other imaging modalities). If findings were not previously known and were considered to harbour potential impact on patient management, they were defined as “unknown incidental findings”.

All patients were clinically followed by reviewing electronic patient charts, to document the impact on patient management of all findings.

### Statistical analyses

Variables were expressed as median and interquartile range (25th, 75th percentiles) or percentages. All statistical analyses were performed using commercially available software (Stata/SE, Version 16.1, StataCorp, College Station, Texas).

## Results

### Patient population

We analyzed 505 PET/CT scans of 162 patients who consented to participate in the VASGRA Cohort Study. After dropping three PET/CT from three patients scanned for the evaluation of mycotic aneurysms prior to graft placements, we finally included 502 PET/CT of 162 patients. Patient demographics of the final study population are displayed in Table 1.

**Table 1:** Patient demographics at the time of the baseline PET/CT.

Number of patients, n	162
Median age in years (IQR)	69 (61–75)
Male gender, n (%)	139 (86%)
Diabetes mellitus^1^, n (%)	27 (17%)
Renal insufficiency^1,2^, n (%)	47 (29%)
Smoking/history of smoking^3^, n (%)	107 (66%)
Fever (≥ 38 °C)^4^, n (%)	90 (56%)
Median C-reactive protein at time of imaging in mg/L^1^ (IQR)	29 (7–82)
Median WBC in G/L^1^ (IQR)	7.2 (5.8–9.7)
Open wound with exposed graft or communicating sinus^1^, n (%)	23 (14%)
Graft insertion in an infected site^1^, n (%)	26 (16%)
**Graft location, n (%)**	
Thoracic	66 (41%)
Thoracoabdominal	4 (2%)
Abdominal/aortoiliacal	73 (45%)
Peripheral	19 (12%)
**Graft material, n (%)**	
PTFE	49 (30%)
PET	41 (25%)
PET, biological graft	16 (10%)
Omniflow	8 (5%)
Other	48 (30%)

*PET/CT* positron emission tomography/computed tomography, *IQR* interquartile range, *WBC* white blood cell count, *PTFE* polytetrafluoroethylene, *PET* polyethyleneterephtalat.

^1^Data of two patients missing.

^2^Defined as glomerular filtration rate < 50 ml/min.

^3^Data of 10 patients missing.

^4^Data of four patients missing.

### Findings

PET/CT was successfully performed with diagnostic image quality after body-weight adjusted intravenous injection of FDG (i.e., 295 Megabecquerel (interquartile range (IQR) 248–360) in all patients with proven or suspected VGEI. One hundred twenty six (78%) patients had a VGEI, as defined by the Management of Aortic Graft Infection Collaboration (MAGIC)^4^. VGEI was ruled out in the remaining 36 patients (22%). In three of these patients (8%), PET/CT was performed after treatment of sepsis, to rule out a VGEI or other remaining infectious foci (all three PET/CT were correctly negative). In another three patients (8%), the suspicion for a graft infection was not confirmed, and the reason for the clinical symptoms, which had led to the study inclusion, remained unclear (i.e. no other site of inflammation of infection was detected). The final diagnosis of the remaining 30 patients with excluded VGEI were pneumonia (n = 9), endocarditis (n = 4), Dressler-syndrome (n = 2), soft tissue infection (n = 3), colitis (n = 2), sternal wound infection and spondylodiscitis (n = 1), thrombophlebitis and spondylodiscitis (n = 1), diverticulitis (n = 1), gastritis (n = 1), pyelonephritis (n = 1), aortic dissection (n = 1), gout (n = 1), retroperitoneal fibrosis with consecutive ureteral obstruction (n = 1), inflammatory aortic aneurysms (n = 1), and sinusitis (n = 1). PET/CT correctly identified 20 (67%) of these findings (pneumonia (n = 9), soft tissue infection (n = 2), colitis (n = 2), sternal wound infection and spondylodiscitis (n = 1), thrombophlebitis and spondylodiscitis (n = 1), diverticulitis (n = 1), gastritis (n = 1), retroperitoneal fibrosis with consecutive ureteral obstruction (n = 1), inflammatory aortic aneurysms (n = 1), and sinusitis (n = 1) (Table 2).

**Table 2:** Unknown and relevant incidental findings in 502 PET/CT scans of 162 patients with proven or suspected vascular graft infections and their impact on patient management.

Location	Findings	Findings with impact on patient management	Type of impact on patient management	Findings without or unknown impact
Brain (n = 1)	Ischemic stroke (1x)	1	Further imaging and treatment	0
Head and neck (n = 22)	Lymph node metastases (recurrence) (1x)	1	Neck dissection	0
	Hypopharyngeal cancer (1x)	1	Radiation therapy	0
	Recurrence of hypopharyngeal cancer (1x)	1	Laryngectomy, neck dissection, chemotherapy	0
	Infected voice prosthesis (1x)	1	Replacement	0
	FDG-avid thyroid lesion (3x)	1	Biopsy = benign	2
	Sinusitis (3x)	1	Endoscopic surgery suggested	2
	Suspected thyroiditis (4x)	2	Hormone therapy (2x)	2
	Suspected tonsillitis (3x)	1	Inspection = confirmed	2
	Asymmetric tonsillar FDG uptake (1x)	0	na	1
	FDG-avid parotid lesion (1x)	1	Biopsy = Warthin tumor	0
	FDG-avid root of teeth (3x)	1	Restoration	2
Chest (n = 53)	FDG-avid lung lesion (4x)	3	follow up = stable (3x)	1
	FDG-negative lung nodule (3x)	3	Resection (1x) = fibrosis; follow up (2x) = stable (1x), decrease (1x)	0
	Growing lung nodule (1x)	1	Resection = cancer	0
	Pleural effusion (6x)	1	Dialysis	5
	Increasing pleural effusion (6x)	0	na	6
	Suspected pleuritis (1x)	0	na	1
	Pneumonia (20x)	4	Antibiotic treatment (4x)	16
	Lung metastasis (2x)	2	Diagnostic wedge resection (1x), imaging follow-up (1x)	0
	Pleural metastasis (1x)	1	Biopsy = confirmed	0
	Esophageal cancer and lymph node metastases (1x)	1	Radiation therapy	0
	Esophagitis (2x)	1	Esophagogastroscopy = confirmed	1
	Aortoesophageal fistula (1x)	1	Esophagectomy	0
	Suspected sarcoid (2x)	0	na	2
	FDG-avid axillary lymphadenopathy (1x)	1	Watch and wait	0
	Unilateral gynecomastia (1x)	0	na	1
	Port misplacement (1x)	1	Removal	0
Cardio vascular system (n = 15)	Vascular graft thrombosis (1x)	1	Prolonged antibiotic treatment	0
	Vascular graft occlusion (1x)	1	PTA	0
	Vascular graft rupture (1x)	1	Endovascular revision	0
	CABG occlusion (1x)	1	Cardiac PET/CT	0
	Suspected mycotic aneurysm (1x)	1	Planned intervention	0
	Abdominal aortic aneurysm (1x)	1	EVAR	0
	Increasing abdominal aortic aneurysm (2x)	1	EVAR	1
	Increasing femoral aneurysm (1x)	0	na	1
	Increasing pericardial effusion (1x)	0	na	1
	Suspected giant cell arteritis (1x)	1	Further work-up	0
	Thrombosis (V. iliaca) (1x)	0	na	1
	Atrial thrombus (1x)	0	na	1
	Suspected subclavian aneurysm (1x)	1	Ultrasound	0
	Endoleak type 2 (1x)	0	na	1
Abdomen (n = 51)	FDG-avid liver lesion (1x)	0	na	1
	FDG-avid pancreatic lesion (1x)	0	na	1
	Suspected cholecystitis (1x)	0	na	1
	Cholestasis (1x)	0	na	1
	Spleen infarction (1x)	0	na	1
	Unclear, focal FDG-avid perianal lesion (2x)	0	na	2
	Unclear, focal FDG-avid colorectal lesion (14x)	11	Coloscopy (9x) = polyp (3x), adenoma (3x), cancer (1x), unclear (1x), fistula (1x), ulcerative colitis (1x); follow up (1x) = diverticulitis	3
	Suspected GIST (1x)	1	Follow up: increasing	0
	Suspected gastritis (3x)	0	na	3
	Suspected colitis (5x)	1	Biopsy = confirmed (1x)	4
	Suspected recurrence of rectal cancer (1x)	0	na	1
	Suspected diverticulitis (1x)	0	na	1
	Psoas abscess (1x)	0	na	1
	Increasing retroperitoneal abscess (2x)	1	Diagnostic puncture	1
	Presacral mass (1x)	1	Biopsy = scar	0
	Hydronephrosis (3x)	3	Double J stent (2x), ultrasound/watch and wait (1x)	0
	Complicated kidney cyst (1x)	0	na	1
	Abdominal lymphadenopathy (1x)	0	na	1
	New (1x) or increasing (2x) ascites	1	na	2
	Unclear, focal FDG-avid prostate lesion (3x)	1	Inspection = prostatitis	2
	Suspected infected kidney cyst (1x)	0	na	1
	Suspected prostatitis and epididymitis (1x)	1	Inspection = confirmed	0
	Unilateral FDG-avid testicle (1x)	1	Ultrasound (no tumor)	0
	Progression of hydronephrosis (1x)	1	Ureteral tumor stent replacement	0
	Recurrence of multiple myeloma (1x)	1	Chemotherapy	0
Bone (n = 30)	Suspected spondylodiscitis (6x)	1	Biopsy = confirmed (1x)	5
	Progressing spondylodiscitis (2x)	1	Change of antibiotic treatment	1
	Suspected infectious arthritis (6x)	1	Arthrocentesis (1x)	5
	Progressing infectious arthritis (1x)	1	Debridement	0
	Metatarsal osteomyelitis (1x)	1	Amputation	0
	Suspected sternal infection (7x), retrosternal abscess (1x)	5	Revision operation (2x), biopsy (3x) = foreign body reaction (1x), confirmed (2x)	3
	Unclear osteolysis (1x)	0	na	1
	Humerus fracture (1x)	1	cast	0
	Vertebral fracture (2x)	0	na	2
	Suspected synovitis (hip) (1x)	0	na	1
	Disseminated metastases (1x)	1	Lymph node biopsy	0
Other (n = 9)	Septic emboli lower limbs (3x)	0	na	3
	Haematoma upper thigh (1x)	0	na	1
	Focal FDG-avid muscle lesion (1x)	0	na	1
	Sub-/cutaneous FDG-avid lesion (3x)	3	Excision (1x) = carcinoma Drainage (2x) = furuncle	0
181		80		101

*Na* not applicable, *FDG* ^18^F-fluorodeoxyglucose, *PTA* percutaneous transluminal angioplasty, *CABG* coronary artery bypass grafting, *PET/CT* positron emission tomography/computed tomography, *EVAR* endovascular aortic repair, *GIST* gastrointestinal stromal tumor.

**Figure 1:**
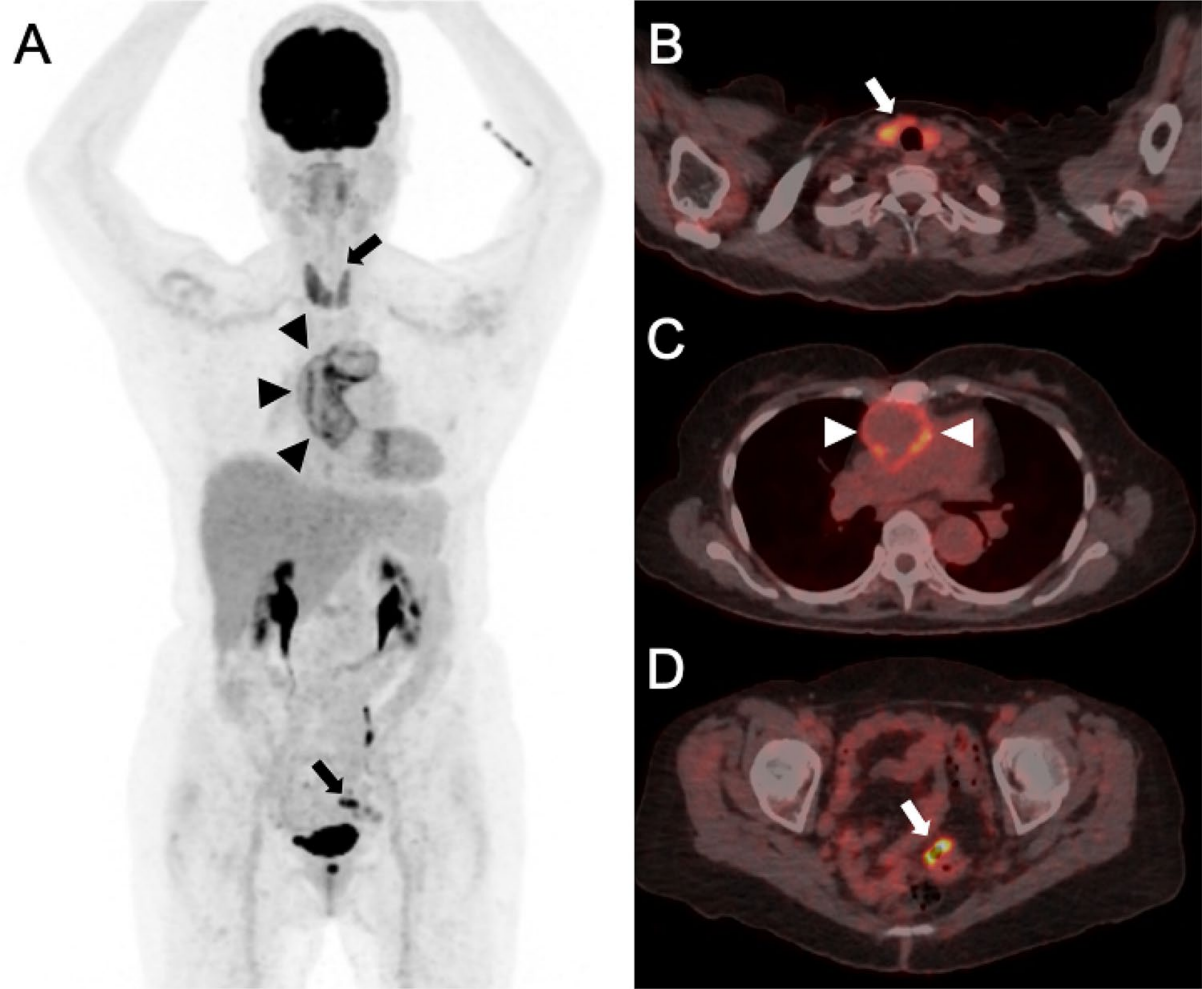
A 70-year old female patient was referred to PET/CT 14 months after the initial diagnosis of a vascular graft infection due to *Streptococcus hominis*. The reason for referral was whether antibiotic treatment could be stopped. PET/CT [maximum intensity reconstructions of PET (**A**) and fused PET/CT images (**B**–**D**)] showed diffuse FDG uptake along the ascendens graft (Index surgery: aortic arch replacement with 28 mm Intergard® prosthesis) (black arrow heads in **A**, white arrow heads in **C**) which was focally pronounced (**A**) indicating persistent infection. Antibiotic treatment was continued for another eight months and then successfully stopped (no signs for recurrence at the last control seven months later). The patient had known thyroiditis, which presented with diffuse FDG uptake (upper black arrow in **A**, white arrow in **B**); this finding was not evaluated for impact on patient management in our study, as it was already known prior to PET/CT. An unknown and relevant incidental finding was detected in the sigmoid colon with intense focal FDG uptake (lower black arrow in **A**, white arrow in **D**). This PET/CT finding was rated to have impact on patient management—subsequent coloscopy and resection revealed a colonic polyp with dysplasia.

**Figure 2:**
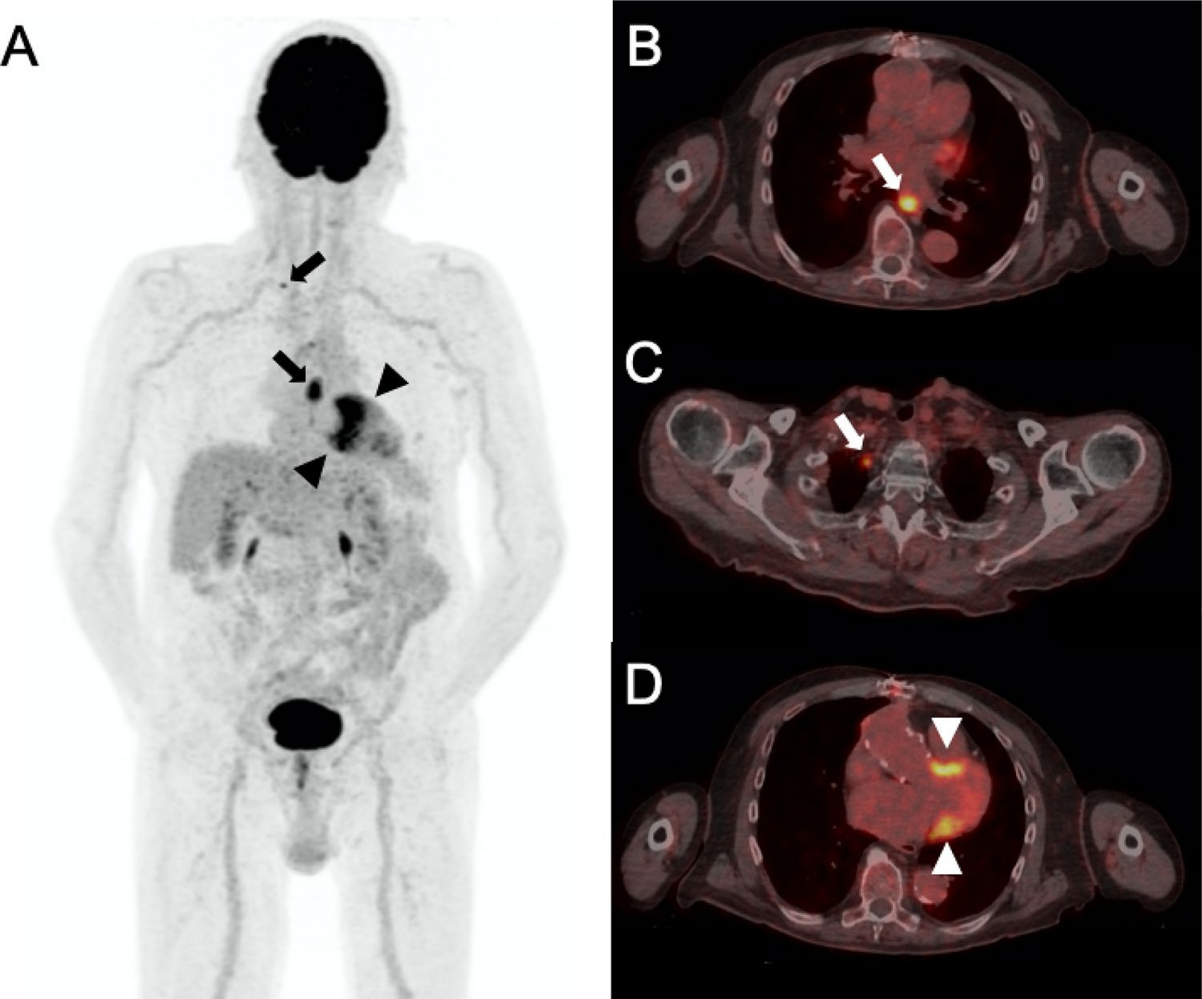
A 75-year old male patient was referred to PET/CT five years and two months after the initial diagnosis of a vascular graft infection due to *Streptococcus pneumoniae*. The reason for referral was whether antibiotic treatment could be stopped. PET/CT [maximum intensity reconstructions of PET (**A**) and fused PET/CT images (**B**–**D**)] showed no focal FDG uptake along the ascendens graft (index surgery: biologic composite graft replacement by Gelweave graft; reconstruction of right pulmonary artery with xenopericardium), indicating complete response to antibiotic therapy. There was physiologic FDG uptake at the base of the left ventricle (black arrow heads in **A**, white arrow heads in **D**). As a relevant incidental finding with impact on patient management, PET/CT detected two foci with intense focal FDG uptake in the esophagus (lower black arrow in **A**, white arrow in **B**) and upper mediastinum (upper black arrow in **A**, white arrow in **C**). Endoscopy and biopsy confirmed metastasized esophageal cancer and the patient was subsequently treated with palliative radiation therapy.

**Figure 3:**
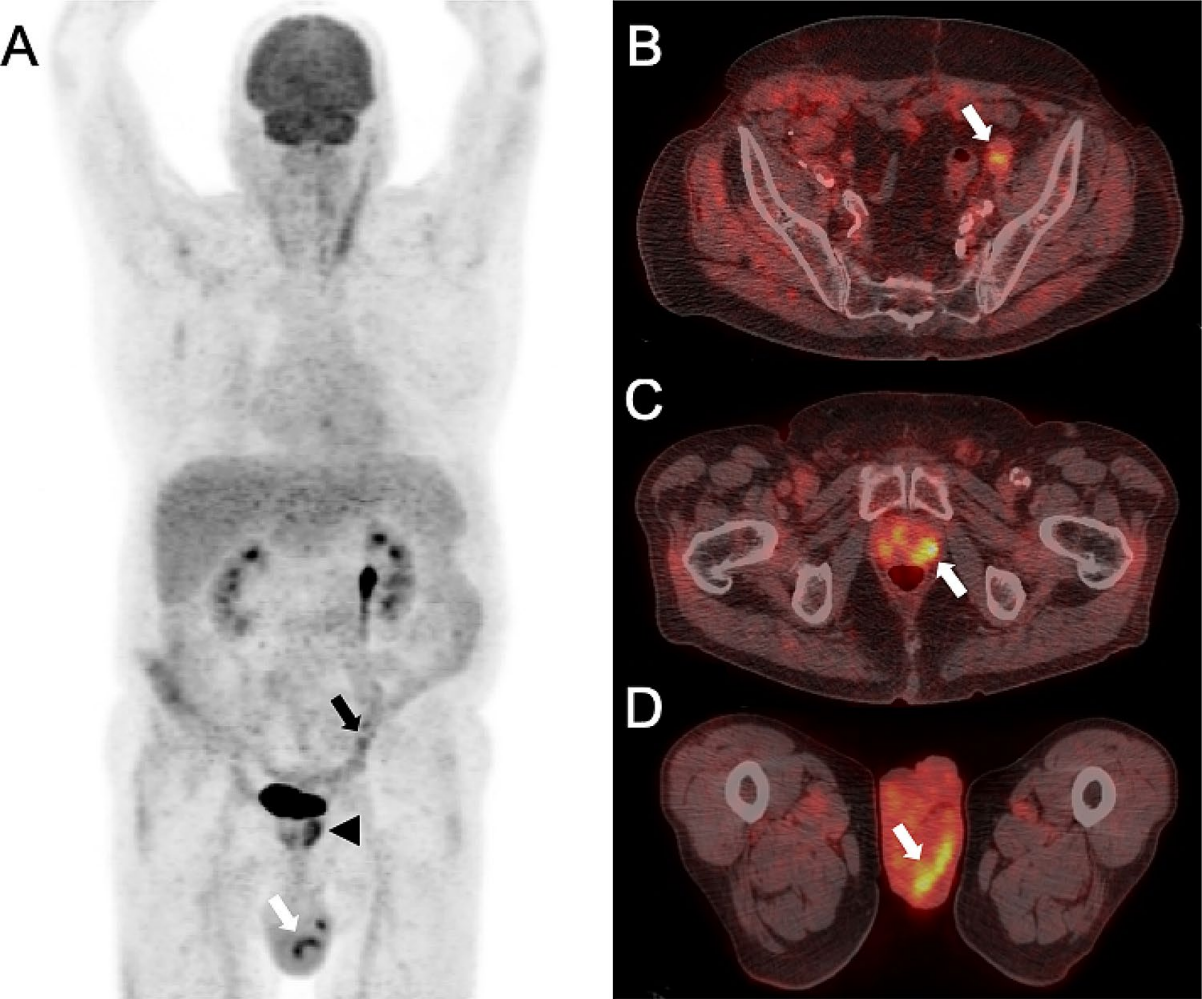
A 61-year old male patient was referred to PET/CT one year and four months after the initial diagnosis of a vascular endograft infection due to *Streptococcus dysgalactiae*. The patient was under antimicrobial therapy, but had acute fever and the reason for referral was the search for new infectious foci. PET/CT [maximum intensity reconstructions of PET (**A**) and fused PET/CT images (**B**–**D**)] showed faint residual focal FDG uptake along the extraanatomical aorto bi-iliac reconstruction (Dacron silver Intergard graft) (black arrow in A, white arrow in **B**), suggestive for persistent infection. Unknown and relevant incidental findings with impact on patient management were detected by PET/CT with intense focal FDG uptake in the prostate gland (black arrow head in A, white arrow in **C**), and in the epididymis (white arrows in **A** and **D**). Subsequent further work-up confirmed prostatitis with *Enterobacter cloacae* and antibiotic treatment was escalated accordingly.

**Figure 4:**
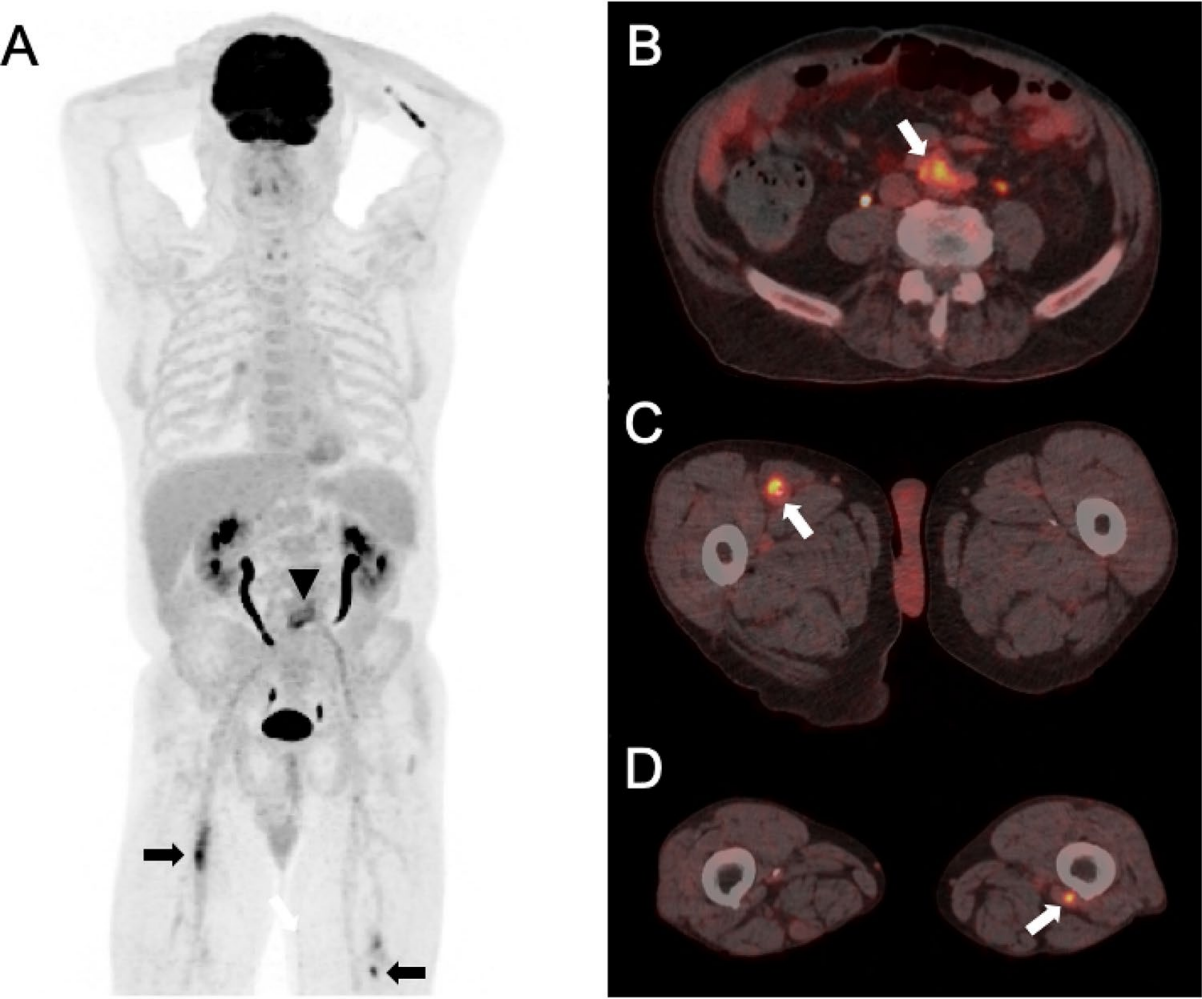
A 66-year old male patient was referred to PET/CT for follow-up of a vascular graft infection with *P. aeruginosa*. PET/CT [maximum intensity reconstructions of PET (**A**) and fused PET/CT images (**B**–**D**)] showed intense focal FDG uptake of the aortobifemoral graft (black arrow head in **A**, white arrow in **B**), suggestive for persistent infection. Subsequently, the aortobifemoral graft was reconstructed in situ with a SilverGuard Dacron graft. An unknown and potentially relevant incidental finding was detected in the upper thighs with intense focal FDG uptake, in line with septic emboli (black arrows in **A**, white arrow in **C** and **D**). The incidental PET/CT finding was rated not to have impact on patient management, since no direct action was initiated with regard to the potential septic emboli in the upper thighs.

Overall, PET/CT identified 181 previously unknown and clinically relevant incidental findings (Figs. 1, 2, 3, 4, Table 2) in 502 scans: 340 PET/CT scans revealed no incidental finding, 147 scans one, 12 scans two, 2 scans three, and 1 scan four incidental findings. The initial PET/CT of each patient revealed 95 findings in 162 examinations (59%), while 86 findings were detected in 340 follow-up examinations. (25%). At least one unknown incidental finding was detected in 162 of 502 (32%) PET/CT scans and in 111 of 162 (69%) patients. Direct impact on patient management was documented in 80 of 181 (44%) of all incidental findings, in 76 of 502 (17%) of all PET/CT scans, and in 59 of 162 (36%) of all patients.

## Discussion

We assessed the rate and the impact on patient management of relevant unknown incidental findings in PET/CT of patients with proven or suspected VGEI.

Our study results show a comparably high rate of relevant unknown incidental findings (181 in 502 examinations), with documented direct impact on patient management in 80 of 181 (44%) of all findings. PET/CT scan- and patient-based evaluation revealed impact on patient management in 76 of 502 (17%) of all PET/CT scans, and in 59 of 162 (36%) of all patients, respectively. Furthermore, PET/CT correctly identified the final diagnosis in 20 of 36 (56%) patients without VGEI.

The present study represents the first study to analyze the impact of incidental findings on patient management in a large prospectively enrolled study population with suspected or proven VGEI. Several other studies have performed similar analysis in different study populations, however, the definitions of “impact on management” and “relevant unknown incidental findings” are very inhomogeneous throughout the literature. For example, Wan et al.^23^ performed PET/CT in patients with psoriasis. Similar to our study, their “relevant” findings were determined by the report of the reading radiologist/nuclear medicine physician. The authors found less relevant findings (i.e. in 12% of 259 patients), and documented that the risk of discovery of significant findings was associated with age. In comparison, the age of our study population was much higher (i.e. 45 years versus 69 years) and we did not exclude patients due to other comorbidities, while Wan et al.^23^ excluded patients with significant comorbidities such as diabetes or uncontrolled hypertension. Both differences may account for the higher incidence of incidental findings in our study.

On the other hand, Nihuis et al.^24^ found a much higher incidence of non-melanoma findings on baseline and annual surveillance CT and PET/CT of asymptomatic melanoma patients (i.e. 912 findings in 1022 scans versus 181 findings in 502 scans in our study). The authors included findings which were not considered “relevant” findings in our study (e.g., coronary artery calcifications or heart valve calcifications), and their rate of findings with impact on patient management was lower as compared to our study (114 of 912 findings versus 80 of 181 findings). The latter, may possibly be explained by the younger age (i.e., mean age 49) of the patient population in the study by Nihuis et al.^24,25^, and also by a higher comorbidity rate in cardiovascular patients, in general^26^.

An even higher rate on impact on management was reported by Hadad et al.^27^, reporting 642 of 1090 (59%) incidental foci to be clinically relevant in a literature review of selected patient cohorts with different types of cancer. We suppose that the comparably lower rate of findings with impact on patient management in our study, may in part be due to systematic differences between patient populations with cancer and patient populations with infectious diseases. For example, an incidentally detected pneumonia in a patient with cancer will result in a change in patient management, as antibiotic treatment will be initiated. However, in a patient with a newly detected VGEI and a synchronous pneumonia, both diseases might be treated with the same antibiotic agent, rendering the incidentally detected pneumonia to “without impact on management”. Hence, we believe that the rate of relevant incidental findings with impact on patient management may even be underestimated in patient populations with infectious diseases.

Finally, PET/CT has been shown to have a high diagnostic accuracy in the detection of VGEI, with an excellent negative predictive value^6,7,28–30^. Our study demonstrates that PET/CT may not only rule out VGEI correctly in patients with suspected VGEI, but may also provide the correct diagnosis in a large proportion of patients without VGEI but with clinical symptoms that were responsible for the referral to PET/CT with the question for VGEI. In fact, PET/CT identified the final diagnosis in 56% of patients with clinical symptoms but without VGEI in our study. Notably, some of the final diagnoses made in the patients without VGEI and with negative PET/CT were diagnoses, which cannot reliably be detected in normal state-of-the-art PET/CT without additional intravenous contrast medium (e.g., pyelonephritis, Dressler-syndrome, or endocarditis). Our data is in line with previous publications indicating that PET/CT is helpful in detecting sites of infections in general [in a median of 54% (range 26–92%)^31^] and is often superior to other imaging modalities, e.g. in fever of unknown origin^13^, chronic osteomyelitis^32^, or mycotic aortic aneurysms^15^.

### Limitations of the study

The definitions of “impact on management” and “relevant unknown incidental findings” are very inhomogeneous throughout the literature and challenge the comparability of our study results to previous ones. To date, our study represents the largest prospectively enrolled study population with suspected or proven VGEI and our results clearly underline the impact of PET/CT on patient management. Notably, the study population is selective and also inhomogeneous (e.g. including aortic and peripheral grafts). Finally, it was beyond the scope of the present study to analyse the risks and drawbacks of the detection of incidental findings, as these findings may cause patient anxiety, trigger further investigations and increase health care costs^33^.

## Conclusion

In proven and suspected VGEI, PET/CT detects a high rate of relevant unknown incidental findings with high impact on patient management.

## Data Availability

The datasets generated during and analysed during the current study are available from the corresponding author on reasonable request.
